# Serological response and protection level evaluation in chickens exposed to grains coated with I2 Newcastle disease virus for effective oral vaccination of village chickens

**DOI:** 10.1186/s12917-016-0785-6

**Published:** 2016-07-25

**Authors:** Reta D. Abdi, Kasahun Amsalu, Olana Merera, Yilkal Asfaw, Eseyas Gelaye, Marta Yami, Teshale Sori

**Affiliations:** Clinical Studies Department, College of Veterinary Medicine and Agriculture, Addis Ababa University, P.O. Box 34, Bishoftu, Ethiopia; Department of Animal Science, University of Tennessee, 2506 River Drive, Knoxville, USA; College of Veterinary Science, Mekelle University, Mekelle, Ethiopia; College of Veterinary Medicine and Agriculture, Samara University, P.O. Box 132, Samara, Ethiopia; National Veterinary Institute, P.O. Box 19, Bishoftu, Ethiopia

**Keywords:** Cereal grains, Chicken, Vaccination, Newcastle disease, Ethiopia

## Abstract

**Background:**

Conventional Newcastle disease (ND) vaccination strategies in village chicken production settings is impractical due to shortage of cold-chain, unsuitability of vaccine administration routes and demanding trained personnel and hence affected its adoption. Results from earlier works elsewhere showed that the heat stable vaccines such as NDI_2_ are thought to be promising for village chickens. This study investigated the suitability and efficacy of Ethiopian cereal grains as carriers for the orally administrated NDI_2_ vaccine in chickens.

**Results:**

Of the 15 treatment groups, drinking water, cracked maize and parboiled barley induced significantly higher HI antibody titer than the other carrier grains and naive control. The higher mean HI titer of chickens in drinking-water, cracked maize and parboiled barley group resulted in 100 % survival rate. In general, there was an inverse relationship between chicken mortality (%) and mean HI titer. Chickens with higher HI antibody titers had better survival rate to the challenge experiment. Booster vaccination at age of day 35 and 105 induced progressively higher HI antibodies titers in all treatment groups.

**Conclusions:**

Vaccine coated parboiled grains could be a good carrier followed by cracked grains while untreated vaccine carrier grains had lower serological responses and protection levels. The current finding gives insights on suitable vaccine delivery system in villages with weak health and transportation infrastructure, unreliable electricity, and minimally trained health workers without catching chickens individually.

## Background

Ethiopia has several diverse indigenous chicken ecotypes. Their diversity is revealed in genetic diversity [1], morphology [2] and production performance [3]. Ethiopia has greater than 42.9 million chickens, with the majority (95 %) kept in village scavenging systems [4]. Chickens in scavenging production systems in rural settings exist with little human input and are constrained by feed, management and disease problems [3]. Newcastle disease (ND) is a devastating disease of both commercial farms and village chickens [5].

In village chickens, different ND virus strains and velogenic pathotypes have been identified in clinically affected and apparently healthy chickens [6]. Therefore, village chickens may serve as a reservoir to disseminate ND virus to the nearby commercial poultry farms [7]. The control of ND in village chickens, therefore, not only reduces the impact of the disease within the village but may also prevent spread to nearby poultry commercial farms. Historically, annual ND outbreaks with high mortality are thought to have deterred the potential of owners to rear village chicken [8]. Effective ND control could, therefore, improve rural farmers’ confidence in the profitability of village chickens production, and in turn play a role in the rural poverty reduction strategy [5]. Vaccination is an effective control strategy against ND [9].

However, the adoption of conventional vaccine strategies in a village production setting faces many challenges. Such challenges include the dependency on cold-chain, large dose preparation per vial and vaccine administration methods (i.e. eye drop and aerosol) developed for a commercial setting [10]. For village chickens, heat stable, non-pathogenic ND strains (I2 and V4) have been identified as an innovate alternatives to traditional vaccines [11]. Heat stable vaccines, such as NDI_2_, are cheaper to produce, do not rely on a cold-chain and can be easily administered with feed grain or water without catching individual bird, and are thought to be suitable and fit for village chickens [12]. The reports from other countries indicated that NDI_2_ vaccine retains potency in the absence of a cold chain, for eight weeks when stored in a cool, dark condition, or at 28 °C in a freeze-dried form [13]. A standard dose of 10^6^EID_50_/bird can protect birds of all age categories when administered via eye-drop, drinking water, certain feeds or injection [13]. Water-based oral delivery system of NDI_2_ vaccine appeared to be constrained by variability in water mineral composition (hard water + high chlorine) at different locations. Hence, carrier grains are preferred to water. Carrier grains adsorb the virus from an aqueous suspension and release it in a viable form in the digestive tract of chickens [13]. Further treatment of grains particularly parboiling could probably remove some deleterious substances from the grain that hampers viability of the I2 virus, thus preserves NDI_2_ [10, 13]. Oral administration of the vaccine, using a common chicken feed as a carrier, is well suited to village chicken production systems.

Feed cultivated in different regions within the same country or different countries have unique characteristic and differ in the capacity to be utilized as vaccine carriers [14]. Therefore, vaccine carriers need to be validated for each local environment. Preliminary work has been undertaken to explore the most suitable carrier grain for the NDI_2_ vaccine in Ethiopia. Although this study led to promising protection [15], only two carrier grains (parboiled barley and sorghum) were investigated despite the large variety of cereal and pulse crops in Ethiopia. Therefore, the current experimental study aimed to determine the suitability and efficacy of five different Ethiopian grains as a carrier for the heat stable NDI_2_ vaccine for oral immunization of rural chickens.

## Methods

### Experimental site

The study was conducted in the College of Veterinary Medicine and Agriculture, Addis Ababa University, Bishoftu, Oromia Regional State, Ethiopia. The College is located 45 km southeast of Addis Ababa at an altitude of 1900 m above sea level. The average annual rainfall is 851 mm and the minimum and maximum temperature is 8.9 °C and 26.2 °C, respectively. The average humidity level is 58.6 %.

### Preparation of experimental house

An experimental house with an area of 16 m × 5 m was constructed. The ceiling, walls and floor of the house were disinfected using 1 % formalin. Clean, disinfected *teff* straw was spread over the floor for bedding. Equipment including drinker, feeder and buckets were cleaned, disinfected and introduced to the houses. The house was kept close for 40 days before the chickens were introduced.

### Experimental management

A total of 400 fertilized Bovans brown chicken eggs were obtained from Genesis Farms PLC and hatched at National Veterinary Institute mini-hatchery room. Eggs were cleaned, fumigated and incubated. Finally, 300 chicks were hatched and collected at the 21st and 22nd day of incubation. Of these, the experimental study utilized 225 chicks.

**Fig. 1:**
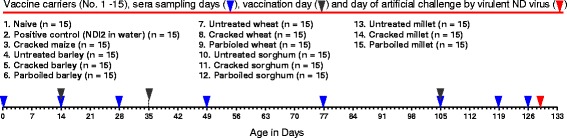
Study design of the experimental NDI2 vaccine trial in Bovans brown chickens. Vaccine carrier groups (experimental units) were listed from No. 1 -15 in this figure. Sera collection was performed across age in days (indicator, blue color) to get 1ml sera per chick at each sampling day from wing vein until day 119. However, for chickens of barely and cracked maize group the last sampling was made on day 126, not on day 119. Vaccination was given at a rate of 107 EID50 NDV I 2 per dose per chick three times (indicator, black color). The value of vaccination by each grain carrier was measured by monitoring antibody production post vaccination in the regularly collected sera and by survival rate of the chickens post artificial inoculation of a virulent local Alemaya strain ND virus at a rate of 0.5 × 106.5 EID50 per chick of all experimental units intramuscularly at breast muscle at day129 (indicator, red color)

The 225 experimental chicks were brooded together until 14 days in a pen with infrared bulbs for heating and *teff* straw for bedding. On the 14th day the chicks were randomly split into the 15 treatment groups as described under study design below (Fig. 1). The chickens were fed on purchased starter commercial ration for 2 months, grower ration the next 3–5 months, and layer ration from 5 months onwards. Water was given *ad libitum*. Antibiotic (oxytetracycline), minerals and vitamins mix in a sachet (i.e. Vytlet) was purchased and supplied for 3 days after each bleeding (Fig. 1). Chickens showing signs of disease (suspected infectious coryza and coccidiosis) were given 20 % oxytetracycline and amprolium. Mortality was recorded daily.

### Experimental design and sample size

Chicken care and experimental procedures were performed under approval from the Animal Ethical Committee (AEC permit No. 6–2010) of the College of Veterinary Medicine and Agriculture of the Addis Ababa University.

A complete randomised design (CRD) was employed. Each chick was identified using wing tag and randomly assigned to one of the 15 pens. Accordingly, a total of 225 chickens were randomly assigned to 15 pens with 15 chicks per pen. The study involved 15 different treatment groups: mock vaccinated (naïve), NDI_2_ vaccine administered in drinking water (conventional delivery as positive control) and NDI_2_ vaccine administered with each of the 13 different carrier grains. The 15 different treatment groups were randomly assigned to the 15 pens as shown on Fig. 1. The sample size per group (*n* = 15 chickens) was calculated by setting type I error at 5 % and type II error at 20 % (80 % power); assumed survival for vaccinated (80 %) and for non-vaccinated chickens (20 %) as described by Chan [16].

### Preparation of carrier grains

All carrier grains were purchased, washed, sun dried and stored at room temperature until use. About 2Kg of each grain was cracked to small size to improve the swallowing by the chicks. About 2 kg of each grain was parboiled grains according to Cumming [17]. The grains were added to boiling water at ratio of 1 kg per 3 l and boiled for two minutes, rinsed with cool distilled water, drained and sun dried.

### Vaccine and coating of grains with the vaccine

Vials of freeze dried NDI_2_ vaccine (300 doses per vial) were purchased from National Veterinary Institute (NVI) located in Bishoftu. Vials were reconstituted in 150 ml of clean, sterile, non-chlorinated distilled water (manufacturer’s instruction). About 48.5 ml of clean, non-chlorinated water was added to the grain first to wet it. Subsequently, for each treatment group (*n* = 15 chickens) 7.5 ml of vaccine suspension (0.5 ml per chick) was mixed with 150 g of carrier grain (10 g per chick) to deliver one dose (10^7^ EID_50_) as described by Wambura et al. [18]. The final suspension of vaccine was stored at room temperature for 6 h. Prior to vaccination, feed was withheld for 7 h, after which the chickens were given the grains coated with the vaccine. Commercial feed was not provided until the chickens consumed the coated grains completely.

### NDI_2_ vaccination interval and sera collection

Vaccination of the chickens was administered on day 14, 35, and 105 as shown on Fig. 1. Sera were collected for haemagglutination inhibition (HI) assay. At every bleeding, sera were collected on day 0, 14, 28, 49, 77, 105 and on day 119 or 126 from the 225 chicken’s wing vein aseptically. Bleeding of maize and barley treatment group transferred to day 126 from 119 due to shortage of syringes and Eppendorf tube for sera storage.

### Haemagglutination inhibition (HI) assay

HI assay was conducted within serology laboratory of NVI. Serum prepared from sequential blood collections (Fig. 1) was heat inactivated at 56 °C for 30 min and stored at −20 °C. The level of ND virus antibodies in serum samples were determined using the HI test as described by OIE [5]. The HI test has 98 % specificity and 69–98 % sensitivity [19]. HI titration was made to determine the right HI concentration via 2-fold serial dilution of 25 μl sera in 25 μl PBS followed by 25 μl loading of viral antigen per well. Then, after 30 min 25 μl of 1 % RBC per well was loaded and kept for 45 min to determine the end point of haemagglutination. The antibody level for each serum sample was expressed as a log to the base two and recorded. For convenience, the titer was recorded as just the log index. For example, the titer of log_2_^2^ was recorded as two. The geometric mean titers (GM) were calculated. In this study we used the published cut off value for the protective HI antibody titer (HI titer ≥ log_2_^3^ i.e. GM ≥ 3) for ND vaccination in chickens [5, 13, 20].

### Experimental challenge by virulent ND virus

All the vaccinated and non-vaccinated chickens were challenged by lethal dose (0.5 × 10^6.5^ ELD_50_ based on viral titration). Local virulent ND Alamya strain was obtained from NVI and inoculated via intramuscular route into the breast muscle. The Alamya strain has a mean embryonic death time of 51.1 h, an intracerebral pathogenicity index of 1.84 and an intravenous pathogenicity index of 2.51 [15]. The challenge time was at the age of 129 which was 3 weeks after the 3rd vaccination.

Post challenge, the chickens were examined daily for 4 weeks until the age of 160 day for clinical signs and death due to ND.

### Statistical analysis

The mean value and standard deviation (SD) of HI antibody titers were determined and classified according to treatment groups. The post vaccination mean HI antibody titers were compared by General Linear model of SPSS version 15. Where the HI results were significant, least square difference (LSD) was used to compare antibody response via pair-wise treatment comparisons. Proportion of chickens with HI antibody titer ≥ log_2_^3^ between treatment groups was used as a cut-off value to compare and decide the protective level [5, 13, 20]. Serology (HI) was to predict level of protection. However, real level of protection was evaluated using challenge experiment. Subsequently, the time to death among treatment groups was compared using Kaplan-Meier survival curve with log rank test used to assess equality of survival distribution among the groups. Chickens were censored in Kaplan-Meier survival analysis whose death was not related to events of interest (i.e. death due to any other causes than ND challenge). Significant differences set at 5 % alpha and at 95 % confidence interval. The relationship between chicken mortality (%) and % of chicken with HI titer above cut-off value were compared to the mean HI titer and statistically tested by Pearson correlation.

## Results

### Serological analysis

#### The base line antibody titer

**Fig. 2:**
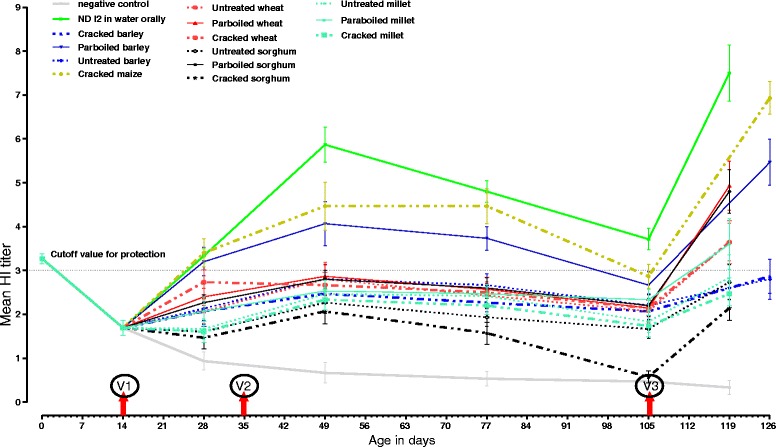
Geometric mean (log2^2^) ± SE of HI antibody titer of chicken vaccinated by coating on 13 different types of grains in comparison to vaccination via water (positive control) and negative control. Red arrows labeled with V1, V2 and V3 on top indicated the day of vaccination. Maternal antibody at day old was high but reduced at day 14. One time vaccination using different carrier grains induced HI titer differently. Second and third round vaccination (booster) upgraded the induction of HI titer significantly (*p*< 0.05) but at varying level depending on the types of grain used as vaccine carrier

The eggs were collected from the same ND vaccinated parents of Bovans brown. The mean maternal antibody (geometric mean ± SD) titer of the 225 study chickens at day old age was 3.3 ± 0.5 and reduced over time during 14 days to 1.5 ± 0.6 at time of vaccination (Fig. 2).

#### Serological response to vaccination

There was a significant variation in the HI response between the different treatment groups (Table 1, Table 2 and Fig. 2). Drinking water, cracked maize and parboiled barley coated with NDI_2_ vaccine induced significantly (*p* < 0.05) higher antibody titers than the other treatment groups. Parboiled sorghum, parboiled wheat, parboiled millet, untreated wheat and cracked wheat coated with the same vaccine induced moderate antibody titers.

**Table 1:** Post vaccination geometric mean (mean ± SD) antibody titers of chickens increased over time by booster vaccination with disparity in titer between vaccine carrier types used

Vaccine carrier type	Post vaccination GM ± SD HI antibody titer (log2) chickens vaccinated by different methods at different ages in days (*N* = 15)					
	Day 28	Day 49	Day 77	Day 105	Day 119	Day 126
Naive	<1^f^	<1^ab^	<1^g^	<1^d^	<1^c^	no
NDI2 in water	3.2 ± 0.6 (15)^a^	5.7 ± 1.6 (15)c	4.7 ± 0.9 (15)^ac^	3.6 ± 0.9 (14)^ab^	7.1 ± 2.4 (14)a	no
Untreated Wheat	1.6 ± 1.0 (15)^b^	2.4 ± 1.5 (15)^d^	1.8 ± 1.3 (15)^bd^	1.7 ± 0.9 (14)^e^	3.3 ± 1.2 (14)^f^	no
Parboiled Wheat	2.3 ± 0.7 (15)^b^	2.6 ± 1.1 (15)^d^	2.1 ± 0.8 (15)^bd^	1.9 ± 0.8 (14)^e^	4.5 ± 2.1 (14)^f^	no
Cracked Wheat	2.3 ± 1.0 (15)b	2.4 ± 1.1 (15)^d^	2.1 ± 1.4 (15)^bd^	2.0 ± 0.7 (14)^e^	3.3 ± 1.8 (14)^f^	no
Untreated Sorghum	1.4 ± 0.6 (15)^f^	2.2 ± 0.8 (15)^d^	1.8 ± 0.8 (15)^bd^	1.4 ± 0.8 (15)^e^	2.4 ± 1.5 (15)^ed^	no
Parboiled Sorghum	2.1 ± 0.7 (15)^b^	2.7 ± 0.8 (15)^d^	2.4 ± 0.9 (15)^bd^	1.5 ± 0.9 (15)^e^	4.4 ± 1.9 (15)^f^	no
Cracked Sorghum	1.2 ± 1.0 (15)^f^	1.6 ± 1.1 (14)^d^	1.1 ± 0.9 (14)^bd^	0.4 ± 0.5 (14)^gb^	1.9 + 1.0 (14)^ed^	no
Untreated Millet	1.3 ± 0.8 (15)^b^	2.4 ± 0.5 (13)^d^	2.3 ± 0.7 (13)^bd^	1.7 ± 0.8 (12)^e^	2.4 ± 1.8 (12)^ed^	no
Parboiled Millet	1.4 ± 1.3 (15)^b^	2.4 ± 0.9 (15)^d^	2.3 ± 0.8 (15)^bd^	2.1 ± 0.9 (15)^e^	3.2 ± 2.0 (15)^f^	no
Cracked millet	1.3 ± 0.5 (15)^f^	1.5 ± 0.6 (15)^d^	1.3 ± 1.0 (15)^bd^	2.1 ± 0.9 (15)^e^	2.5 ± 0.8 (14)^ed^	no
Cracked Barely	1.6 ± 1.2 (15)b	2.4 ± 0.6 (15)^d^	1.7 ± 0.9 (15)^bd^	1.5 ± 0.9 (15)^e^	no	2.5 ± 1.4 (15)^ed^
Parboiled barely	3.0 ± 1.3 (15)^a^	3.7 ± 1.9 (15)^ab^	3.6 ± 1.0 (15)^e^	2.4 ± 1.2 (15)^fc^	no	5.1 ± 2.0 (15)g
Untreated Barely	1.7 ± 1.0 (15)b	2.5 ± 1.3 (15)^d^	2.4 ± 1.0 (15)^bd^	2.0 ± 1.0 (15)^d^	no	2.8 ± 1.8 (15)^ed^
Cracked Maize	3.2 ± 1.2 (15)^a^	4.2. ± 2.1 (15)^ab^	3.9 ± 1.6 (15)^ac^	2.7 ± 1.1 (15)^fc^	no	6.8 ± 1.4 (15)g

Means with the same letters in the same column are not significantly different at 0.05 confidence level, “no” = sera not collected

**Table 2:** GLM least square difference for pair-wise comparison of HI titers among 15 different vaccine carrier grain types at day 28 (below diagonal), day 126 for barely and maize and day 119 for the rest grains (above diagonal) for significance test at 95 % CI

Grain	1	2	3	4	5	6	7	8	9	10	11	12	13	14	15
1	-	0.000	0.000	0.000	0.000	0.000	0.000	0.000	0.000	0.000	0.000	0.000	0.000	0.000	0.000
2	0.000	-	0.000	0.000	0.000	0.000	0.000	0.000	0.000	0.000	0.000	0.000	0.358	0.001	0.000
3	0.003	0.000	-	0.04	1	0.14	0.06	0.017	0.215	0.945	0.056	0.208	0.000	0.003	0.172
4	0.000	0.012	0.247	-	0.04	0.000	0.835	0.000	0.001	0.031	0.000	0.001	0.001	0.383	0.001
5	0.000	0.082	0.054	0.44	-	0.14	0.06	0.017	0.215	0.945	0.056	0.208	0.000	0.003	0.172
6	0.062	0.000	0.271	0.023	0.002	-	0.001	0.338	0.876	0.152	0.66	0.826	0.000	0.000	0.912
7	0.000	0.003	0.463	0.656	0.218	0.062	-	0.000	0.002	0.048	0.000	0.001	0.000	0.271	0.001
8	0.119	0.000	0.177	0.012	0.001	0.783	0.035	-	0.29	0.018	0.599	0.24	0.000	0.000	0.286
9	0.031	0.000	0.516	0.078	0.012	0.692	0.173	0.516	-	0.233	0.568	0.959	0.000	0.000	0.959
10	0.002	0.000	0.855	0.32	0.075	0.192	0.576	0.119	0.404	-	0.061	0.226	0.000	0.002	0.187
11	0.062	0.000	0.271	0.023	0.002	1	0.062	0.783	0.692	0.192	-	0.509	0.000	0.000	0.582
12	0.002	0.000	0.855	0.32	0.075	0.192	0.576	0.119	0.404	1	0.192	-	0.000	0.000	0.912
13	0.000	0.906	0.000	0.008	0.059	0.000	0.002	0.000	0.000	0.000	0.000	0.000	-	0.015	0.000
14	0.000	0.666	0.001	0.034	0.182	0.000	0.009	0.000	0.000	0.002	0.000	0.002	0.576	-	0.000
15	0.001	0.001	0.714	0.417	0.11	0.136	0.709	0.082	0.312	0.852	0.136	0.852	0.000	0.003	-

Keys: 1 = Naïve; 2 = ND I2 in water; 3 = Untreated wheat; 4 = Parboiled wheat; 5 = Cracked wheat; 6 = untreated sorghum; 7 = Parboiled sorghum; 8 = Cracked sorghum; 9 = Untreated millet; 10 = Parboiled millet; 11 = Cracked millet; 12 = Cracked barley; 13 = Cracked maize; 14 = Parboiled barley; 15 = Untreated barley

The pair-wise GLM least square difference significant level was conducted but not included for day 49, 77 and 105 of the HI assay in this document

After one booster vaccination (i.e. day 14 and 35), the chickens in most of the treatment groups had a mean HI titer between (log_2_^2^) and (log_2_^3^) until day 105. The administration of the 2nd booster at day 105, further increased HI titer significantly. Therefore, not only the 1st booster (day 35) but also the 2nd booster (day 105) vaccination increased the induction of HI antibody progressively as shown in Table 1 and Fig. 2.

Three of the 15 treatment groups had the HI titer above log_2_^3^ throughout the study until day 105. The assay on day 119 indicated that eight of the treatment groups had HI titer above log_2_^3^. This implies that the 2nd booster vaccination (day 105) induced higher HI antibody in chickens of five more treatment groups (HI > log_2_^3^). NDI_2_ in water, cracked maize and parboiled barley had significantly (*p* < 0.05) higher HI titer than the others groups but no significant difference was observed among three of them (*p* > 0.05). ND I2 vaccine coated parboiled wheat and parboiled sorghum had comparable antibody titer of log_2_^4.5^ and log_2_^4.1^, respectively. Throughout the study period ND I2 coated cracked sorghum induced lower HI antibody response (Table 1, Table 2 and Fig. 2).

#### Percentage of chickens with HI titer above log_2_^3^ (cut-off value for protection)

The percentage of chickens with a protective level (HI titers ≥ log_2_^3^) of antibodies varied with the type of vaccine carrier grain used and frequency of vaccinations (Table 3).

**Table 3:** The percentage of chickens with HI titers above the protective titer (≥ log_2_2^3^) related to the types of vaccine carrier used

	Number of chickens (%) with HI log2) ≥ 3.0 of all chicks (*N* = 15)					
Vaccine carrier type	Day 28	Day 49	Day 77^¥^	Day 105	Day 119	Day 126
Naive	0(0.0)	0(0.0)	0(0.0)	0(0.0)	0(0.0)	no
NDI2 in water	12(80)	15(100)	15(100)	10(66.7)	15(100)	no
Untreated Wheat	7(46.7)	8(53.3)	6 (40)	5(33.3)	10 (71.4)	no
Parboiled Wheat	8(53.3)	11(73)	7(46.7)	5(35.5)	11(78.5)	no
Cracked Wheat	7(46.7)	9(60)	5(33.3)	4(28.5)	9 (60)	no
Untreated Sorghum	1(6.6)	6(40)	4(26.7)	2(13.3)	7(46.7)	no
Parboiled Sorghum	6(40)	8(53.3)	6(40)	5(33.5)	9(60)	no
Cracked Sorghum	2(13.3)	5(33.3)	2(13.3)	0(0.0)	3(20)	no
Untreated Millet	4(26.7)	6(50)	5(41.7)	3(25)	7(58.3)	no
Parboiled Millet	8(53.3)	10(66.7)	7(46.7)	5(30)	11(73.3)	no
Cracked millet	4(26.7)	7(46.7)	5(33.3)	3(30)	8(53.3)	no
Cracked Barely	7(46.7)	9(60)	6(40)	4(26.7)	no^a^	8(53.3)
Parboiled barely	10(66.7)	12 (75)	8(53.3)	9(60)	no	13(86.7)
Untreated Barely	6(40)	9(60)	5(33.3)	6 (40)	no	8 (53.3)
Cracked Maize	11(73)	13(86.7)	10(66.7)	9(60)	no	15(100)

^a^no = represented sera not collected. Figures outside the brackets indicated number of chickens in each group having HI titer above cut off value whereas figures in brackets indicated percentages

Following the 1st vaccination, HI titer of ≥ log_2_^3^ was detected in 80 % of chickens vaccinated via conventional (water) and cracked maize. It was 66.7 % in the parboiled barley treatment group and 50 % in the parboiled wheat, cracked wheat, untreated wheat and parboiled millet treatment groups. The percentage of chickens’ with HI titer ≥ log_2_^3^ was less in the cracked sorghum and untreated sorghum coated treatment groups (Table 3). After the first booster vaccination, there was an increase in the proportion of chickens with HI titer above (> log_2_^3^) in comparison to the first vaccination. The highest percentage was observed in chicken vaccinated via conventional method (100 %), followed by cracked maize (86.7 %), parboiled barley (75 %) and parboiled wheat (73 %) in that order, as shown in Table 3. The cracked sorghum and untreated sorghum coated with vaccine had lowest titer but they scored better than their titer induced during the first vaccination. After the 2nd booster vaccination, all (100 %) of the vaccinated chickens via conventional (water) and cracked maize had HI titer ≥ log_2_^3^. In the naive control chicken, none (0 %) of the chickens had HI titer ≥ log_2_^3^ throughout the study period as shown in Table 3.

### Monitoring the survival rate of chickens following artificial challenge

**Fig. 3:**
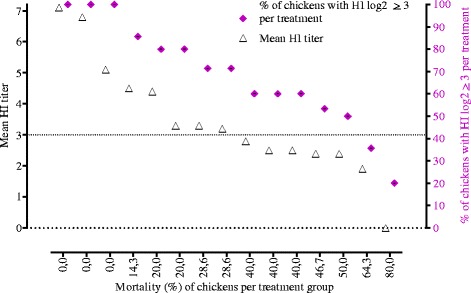
The inverse relationship between chicken mortality rate (%) vs. mean HI titre (left y-axis) and % of chicken with HI titre above cut-off value (right y-axis). Each bullet points represented each treatment group of the 15 treatments. Each bullet points represents each treatment group of the 15 treatments

The mean HI titer and mortality (%) were inversely correlated as shown on Fig. 3 (*r* = −0.938, *p* < 0.000). The mean HI titer above log_2_^5^ corresponds to a 100 % survival rate as shown in chickens vaccinated by conventional (water), cracked maize and parboiled wheat coated vaccine. The HI titer between log_2_^(3–5)^ corresponded to survival rates between 70–80 %. The HI titer between log_2_^2^ – log_2_^3^ corresponded to survival rates of 35–60 % as shown in chickens vaccinated by cracked sorghum, untreated millet, untreated sorghum, cracked millet and cracked barley coated vaccine (Table 4, Fig. 3, left y-axis).

**Table 4:** Dependency of morbidity, mortality and survival rate of chickens on types of vaccine carrier used to resist virulent strain ND virus challenge after three times vaccination

Vaccine carrier type	Chicks N†	Mean HI titer	# morbidity (%)	# death total	#mortality^a^ (%)	Lower 95 % CI	Upper 95 % CI	# Survival (%)
NDI2 in water	15	7.1	0(0.0)	0	0(0.0)	0	0	15(100)
Cracked maize	15	6.8	0 (0)	0	0(0.0)	0	0	15(100)
Parboiled barley	15	5.1	0(0.0)	0	0(0.0)	0	0	15(100)
Parboiled wheat	14	4.5	5(30.0)	2	2(14.3)	0	32.6	12(85.7)
Untreated wheat	15	3.3	6 (40.0)	3	3(20.0)	0	40.2	12(80.0)
Parboiled sorghum	15	4.4	6(26.7)	3	3(20.0)	0	40.2	12 (80.0)
Cracked wheat	14	3.3	8(53.3)	4	4(28.6)	4.9	52.2	10(71.4)
Parboiled millet	14	3.2	7(46.7)	4	4(28.6)	4.9	52.2	10(71.4)
Cracked millet	15	2.5	10(66.7)	6	6(40.0)	15.2	64.8	9(60.0)
Untreated barley	15	2.8	8(53.3)	6	6(40.0)	15.2	64.8	9(60.0)
Cracked barley	15	2.5	7(46.7)	6	6(40.0)	15.2	64.8	9(60.0)
Untreated sorghum	15	2.4	10(66.7)	7	7(46.7)	21.4	71.9	8(53.3)
Untreated millet	12	2.4	8(53.3)	6	6 (50)	21.7	78.3	6 (50.0)
Cracked sorghum	14	1.9	11(73.3)	9	9(64.3)	39.2	89.4	5(35.7)
Naïve	15	0.0	14 (93.3)	12	12(80.0)	59.8	100	3 (20.0)

N^†^ represented number of chickens present in each group at age of 129 day (onset of the challenge). Figures outside the brackets indicated number of chickens in each group whereas figures in brackets indicated percentages. ^a^ Chickens in the treatment groups listed up to parboiled millet had significantly (*p* < 0.0001) higher survival rate compared to the naïve group. The lower and upper 95 % confidence interval (CI) of mortality of chickens per each treatment group was shown in the table

Figure 3 (right y-axis) indicated that when more than 80 % of the chickens in a treatment group had HI titer above cut-off value, the mortality (%) in that treatment group was nearly 0 %. In parboiled wheat, the mortality (%) was nearly 15 % while 78.5 % of the chickens in this treatment group had HI titre above cut-off value for protection. In this study, more than 50 % of the chickens in majority of the treatment groups had HI titre above cut-off value for protection that that subsequently resulted in mortality below 50 %. However, in cracked sorghum group mortality was higher (i.e. 64.3 %) as only 20 % of the chickens had HI titer above the cut-off value.

### Survival analysis

**Fig. 4:**
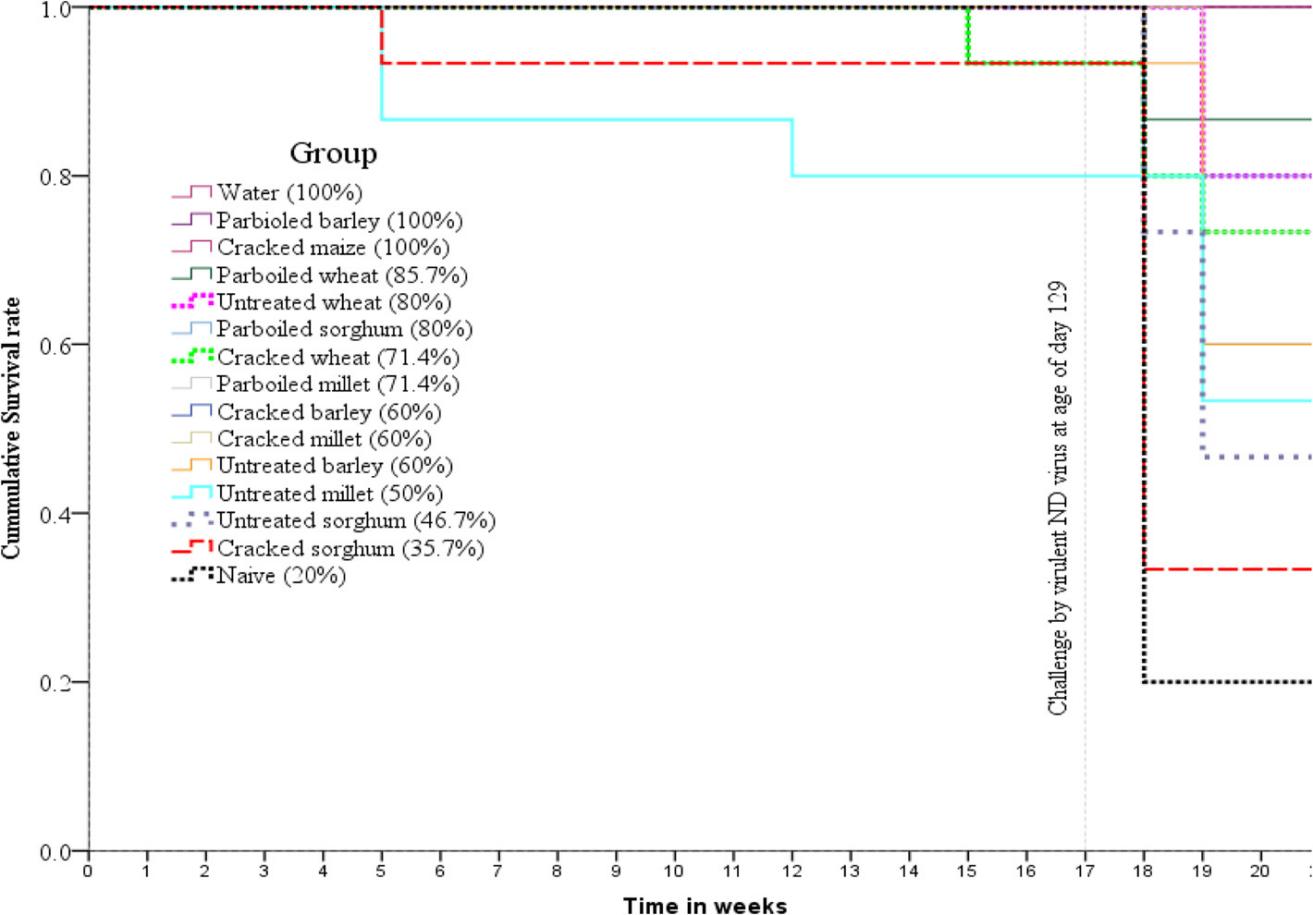
Kaplan-Meier survival analysis of chickens vaccinated by NDI2 via different grain carriers as assessed before and after ND virus challenge by virulent strain at age of day 129. Morbidity was started on day 3 and severe mortality prevailed up to 14 days post challenge during 30 days of follow up. Vaccination using any type of vaccine carrier grain had an impact better than the naïve for survival rate of the chickens. However, different vaccine carrier grains had different survival rates in the range of 35.7 - 100% whilst 20% in naïve. The survival rate was significantly different among the treatment groups (Log-rank = 77.3498, D.F. = 14, p < 0.0000). For example, oral vaccination using water, parboiled barley and cracked maize induced 100% survival until end of the experimental period i.e. week 21

The overall differences in mortality (%) were statistically significant (Log-rank = 77.35, D.F. = 14, *p* < 0.0000) among the treatment groups (Fig. 4). A small number of chickens from untreated (*n* = 3) and parboiled millet (*n* = 1), cracked sorghum (*n* = 1), parboiled (*n* = 1) and cracked wheat (*n* = 1) died due to other disease than ND at different days during the study. Infectious cryza and coccidiosis were suspected and treated long ago (about 2 months) before the inoculation of the velogenic ND viral challenge. The dead chickens were censored in Kaplan-Meier survival analysis as their death was happened before the inoculation of the virulent ND virus challenge. Post-challenge with ND virus, mortality occurred only during the first 14 days; no mortality was observed during days 15 to 30 of the follow up period (Table 4; Fig. 4). The death of chickens due to the challenge started at fourth days post challenge and persisted up to 13th days with the median survival time of 7 days among the dead chickens by the challenge.

## Discussion

The control of ND in village chickens can make a vital contribution to the improvement of household food security and poverty reduction in Ethiopia. Intensive commercial poultry farmers in Ethiopia vaccinate chickens routinely, but village chicken farmers do not [3]. In the current study, five cereal grain species, in 3 different forms, were evaluated for suitability and efficacy as a carrier for the NDI_2_ vaccine as a way forward for developing suitable vaccine delivery system for village chicken production system. The carrier grains have been shown to adsorb the virus from an aqueous suspension and release it in a viable form in the digestive tract of chickens [13]. However, the virus adsorption and releasing capacity of cereal grains varies among grain species and forms of preparation [13]. Grain based vaccine efficacy could be assessed via (i) monitoring sero-conversion, (ii) post vaccination challenge and (iii) survival rate as recommended by Spradbrow [13, 21, 22]. In this study, five different cereal grains were evaluated for their suitability and efficacy as vaccine carrier using the above mentioned three assessment methods.

### Maternal HI antibody titer

At day old, chicks included in this study have HI antibody titer above log_2_^3^. Such high maternal antibody titer in the baby chicks is deleterious to vaccination [5]. Thus, we waited until it declined to log_2_^1.5^ titer at 14 days to overcome the risk of its interference with the vaccine. In line with this, it has been well established that chicks from immunized parents possess high level of maternal antibody which protects the chicks against virulent virus and interferes with vaccine antigens [23, 24].

### Monitoring serological response using HI antibody titer

An HI titer of ≥ log_2_^3^ following vaccination has been considered protective against virulent ND virus. HI titers lower than log_2_^3^ have been associated with lower levels of protection [13, 20]. The results of the current study showed that administration of 2nd and 3rd booster vaccination significantly and progressively increased HI antibody titer in all treatment groups except the naïve control. Furthermore, different grains induced different level of HI antibody titer. This implies the presence of inherent variation in virus carrying capacity of different grains. This is an opportunity to screen grains of different species and varieties. Interestingly, treating grains (either cracking or parboiling) increased their efficacy as vaccine carrier, evident by induction of higher HI antibody titer than that was induced by untreated form. Similar results have been reported in Nigeria by Olabode [25] as to the efficacy of treated grain particularly maize compared to untreated grain. Grains have been known to contain tannins, anthraquinone, cardiac glycosides and alkaloids. Some of these chemicals have been shown to have antiviral properties [22, 26]. The higher HI titer induced by treated grains than untreated ones could be due to the fact that cracking grains increase the surface area of the grains to adsorb the vaccine virus [10, 13, 14, 18, 22, 25, 26]. Likewise, parboiling might destroy the antiviral factors from the seed of the grains [22, 26]. Hence, both cracking and parboiling grains would induce better HI antibody titer via adsorbing and releasing live virus in the gut. However, inconsistent results were observed for sorghum in which cracked, untreated and parboiled sorghum induced HI titer of ≥ log_2_^3^ in 20 %, 46.7 % and 60 % of chickens, respectively, after the 3rd booster vaccination.

This implies that repeated vaccination induces progressively higher HI titer that could correspond to high levels of protection. Moreover, different grain species as well as their preparations in different forms have induced different level of HI antibody titers. Treatment of grains (parboiling or cracking) renders the grains suitable for vaccine carrier for oral vaccination as evident from their corresponding improved antibody response. Higher HI antibody titer corresponded to higher protection level for most grain carriers in the order of parboiled, cracked and untreated grains. This, however, is not the case for sorghum particularly untreated form was found to be better than cracked form in HI titer and post challenge survival. Exceptionally, the parboiled and untreated sorghum induced higher HI titer than the cracked sorghum. Cracking in sorghum might have released anti-viral factors (tannins) and enzymes that probably inactivated the virus. To this end anthraquinone, alkaloids and cardiac glycosides were reported to be abundant in sorghum [26].

Despite low HI antibody titer induced by some grains (sorghum), unexpectedly higher survival % was observed following the challenge. This perhaps highlights the weak correlation between low HI titer and protection for sorghum and the difficulties of using serum antibodies to determine protection to respiratory pathogens [27, 28]. In addition to serum antibody, secretory antibody (IgA) at mucosal surfaces and cell mediated immunity are thought to play a role in resistance to challenge [29]. In general, however, this study showed a negative correlation between mean HI titer and mortality % at the group level (Fig. 3).

Other than the treatment (grain type for vaccine delivery) that of the pen effect has been handled by grouping of the chickens by randomization. However, pens are not replicated due to shortage of space in the experimental house and finance to account for the pen effect on immune response. We believe that the variance among pens is generally less than the variance of chickens within pens due to treatment effect.

### Protection assessment following artificial viral challenge

In the current study, intramuscular route of the breast muscle was used to challenge the chickens. Intramuscular route was preferred as it allows birds to receive equal doses of the challenge virus. Water, cracked maize and parboiled barley were found to be carriers of NDI_2_ vaccine than the others. They protected 100 % (15/15) of the chickens against virulent challenge with ND strain compared to 20 % protection in the naïve group (3/15). In agreement with this results of full protection (100 %) using the NDI_2_ vaccination via drinking water and parboiled barley was previously reported in Ethiopia [15]. Another similar heat stable vaccine (NDV_4)_ coated cracked maize has also been reported to induce high levels of protection in Nigeria [25] which is similar to our NDI_2_ results. The 80 % protection achieved using parboiled sorghum currently is, however, in disagreement with previous results report in Ethiopia [15], Nigeria [30] and Tanzania [31].

It is widely accepted that the recommended protection level of ND vaccination is 80 % [32]. However, we have achieved 100 % protection using NDI_2_ in water, cracked maize and parboiled barley at on-station condition.

The survival rate for the chickens vaccinated with untreated and cracked millet is 60 %, but 73.3 % for the parboiled millet. Our current finding is comparable to the 70 % survival rate reported elsewhere among chicken vaccinated with NDI_2_ using parboiledand broken millet [33].

The chicken survival rate is 71.4 % for cracked wheat, 80 % for untreated wheat and 85.7 % for parboiled wheat group. The untreated barley as carrier for NDI_2_ in the current study had 60 % survival rate. This is far lower than the previous work in Ethiopia [15] and what was reported elsewhere with 100 % protection [34]. This variation in serological response between and within grain forms could be explained by (i) differences in the contents of anti-viral factors in the grains, (ii) variability in vaccine carrying capacity among different grains, (iii) difference in agro-ecology and soil characteristics that can have different effect on physico- chemical characteristics of the grains. Oakeley [22] suggested that grains grown in different agro-ecology and on different soil characteristics tend to vary in their vaccine virus carrying capacity due to variation in the grains’ physico- chemical characteristics, especially their surface properties and chlorine content [22].

In general, parboiled grains, followed by cracked ones, induced higher serological response and protection level than intact (untreated) grains. Heating, soaking, washing and cracking grains might be useful in developing a successful vaccine carrier feed. Similar findings have been reported from other countries [17, 35]. Cracked maize and parboiled barley are found to be better vaccine carriers under Ethiopian context. Our promising finding on wheat was consistent with Spradbrow [13]. Thus, cracked maize, parboiled barley or parboiled wheat should be the base for a large scale grain screening and oral based ND vaccination program. They can be used widely as carrier for oral NDI_2_ vaccination. However, it should be noted that the protection level of the grain based NDI_2_ vaccine varies under laboratory conditions i.e. > 90 % protection [36] and under real village conditions i.e. < 60 % [37] and with vaccine delivered by farmers [36]. This signals the necessity of pilot field trial at village level to evaluate the results of the current on-station study at real village conditions.

### Durability of the virus in the grains and at room temperature

In the current study, the vaccine virus coated on grains was highly immunogenic after 6 h of exposure to room temperature; hence it could be used to vaccinate village chicken against ND. We haven’t measured the upper limit of the time when it is still efficacious in order to enable central vaccine production and then distribution to rural villages. A very important condition for successful development and use of any chosen feed as vaccine carrier is the ability to allow firm binding or adherence of the coated vaccine virus without interfering with the survival of the vaccine virus. In this regard different results have been reported from different countries. According to Tu et al. [12], the NDI_2_ in grains has substantial infectivity and induction of immunity in chickens under laboratory conditionsafter storage for 17 days and in village conditions after storage for 21 days. Echeonwu et al. [30] reported that the virus coated feed without additive remained stable and immunogenic for 3 weeks (millet); 3.5 weeks (sorghum) and 5 weeks for maize at room temperature. Nassir et al. [15] recovered viable vaccine virus after 14 h at room temperature on parboiled barley.

## Conclusion

Chickens fed on NDI_2_ vaccine coated grains induced higher HI antibody than the naïve control and in most cases protected chickens from virulent ND virus challenge. Significant variation was detected between the types and forms of grains and the use of booster vaccinations. Cracked maize, parboiled barley, untreated and parboiled wheat in addition to parboiled sorghum would be promising suitable carriers for large scale administration of NDI_2_ vaccine under Ethiopian field (village) conditions. Procedures of an on-going vaccine efficacy evaluation would be needed using grain carriers under field conditions. In addition, region based large scale grain screening using cracked maize, parboiled barley and parboiled wheat as a reference for oral based ND vaccination program is suggested.

## Abbreviations

EID, embryo infective dose; ELD, embro lethal dose; GM, geometric mean titers; HI, heamagglutination inhibition; LSD, least square difference; ND, newcastle disease; NVI, national veterinary institute; OIE, world organization for animal health; PBS, phosphate buffered saline; RBC, red blood cell
