# Erratum to: Serological response and protection level evaluation in chickens exposed to grains coated with I2 Newcastle disease virus for effective oral vaccination of village chickens

**DOI:** 10.1186/s12917-016-0909-z

**Published:** 2016-12-08

**Authors:** Reta D. Abdi, Kassaw Amssalu Tadesse, Olana Merera, Yilkal Asfaw, Eseyas Gelaye, Marta Yami, Teshale Sori

**Affiliations:** 1 Clinical Studies Department, College of Veterinary Medicine and Agriculture, Addis Ababa University, P.O. Box 34, Bishoftu, Ethiopia; 2 Department of Animal Science, University of Tennessee, 2506 River Drive, Knoxville, USA; 3 College of Veterinary Science, Mekelle University, Mekelle, Ethiopia; 4 College of Veterinary Medicine and Agriculture, Samara University, P.O. Box 132, Samara, Ethiopia; 5 National Veterinary Institute, P.O. Box 19, Bishoftu, Ethiopia

## Erratum

The original article [1] contains an error in the author list that was carried forward by the production team handling this article.

The co-lead author, Kassaw Amssalu Tadesse’s name is incorrectly displayed as ‘Kasahun Amsalu’; this should instead be displayed as ‘Kassaw Amssalu Tadesse’.
